# Comments on “Probiotic *Bifidobacterium* strains and galactooligosaccharides improve intestinal barrier function in obese adults but show no synergism when used together as synbiotics”

**DOI:** 10.3389/fphar.2023.1228359

**Published:** 2023-08-17

**Authors:** Yazan Ranneh, Ayman M. Mahmoud, Abdulmannan Fadel, Mohd Fadzelly Abu Bakar, Abdah Md Akim

**Affiliations:** ^1^ Department of Nutrition and Dietetics, College of Pharmacy, Al-Ain University, Al-Ain, United Arab Emirates; ^2^ Zoology Department, Faculty of Science, Physiology Division, Beni-Suef University, Beni-Suef, Egypt; ^3^ Department of Life Sciences, Faculty of Science and Engineering, Manchester Metropolitan University, Manchester, United Kingdom; ^4^ School of Sport and Exercise Sciences, Liver-Pool John Moores University, Liverpool, United Kingdom; ^5^ Faculty of Applied Sciences and Technology, Universiti Tun Hussein Onn Malaysia (UTHM), Pagoh, Johor, Malaysia; ^6^ Department of Biomedical Sciences, Faculty of Medicine and Health Sciences, Universiti Putra Malaysia, Serdang, Selangor, Malaysia

**Keywords:** lipopolysaccharide, serum, *Bifidobacterium*, lipopolysaccharide binding protein, obesity

## Introduction

The development of obesity is influenced by various factors; however, in recent decades, researchers have been particularly intrigued by the role of gut microbiota. Specific changes in bacterial taxa and a decrease in diversity within the gut microbiota are observed among obese individuals (Hou et al., 2017). These changes contribute to an elevation in intestinal permeability and translocation of lipopolysaccharide (LPS) into blood circulation, leading to metabolic endotoxemia (Mohammad and Thiemermann, 2021). LPS has been proven to increase tight junction permeability by inducing enterocyte membrane expression and localization of Toll-like Receptor-4 (Guo et al., 2013). Obesity has been linked to elevated intestinal permeability. In genetically obese mice, there is an upregulation of intestinal permeability along with an increase in circulating endotoxins and pro-inflammatory cytokines such as interleukin one beta (IL-1β), interleukin-6 (IL-6), interferon-gamma (INFγ), and tumor necrosis factor (TNF-α), when compared to wild-type mice (Lee et al., 2018). Conversely, obesity induced by a high-fat diet, also known as diet-induced obesity (DIO), is associated with alterations in the gut microbial population that are associated with inflammation and heightened intestinal permeability, likely due to diminished expression of tight junction (TJ)-related genes, including ZO-1 and occludin. These collective observations indicate that obesity-related inflammation may be linked to disruptions in the integrity of TJ structures and alterations in the composition of the intestinal microbiota (Aleman et al., 2023).

Obese individuals have shown higher serum LPS concentrations along with TNF-α and IL-6. Among Mexican obese subjects, a positive correlation has been found between serum LPS and BMI, triglyceride, and waist circumference (Radilla-Vázquez et al., 2016). Thus, the assessment of serum endotoxin concentrations along with cytokines profile indicates the degree of systemic inflammation in obese individuals. Subsequently, several clinical trials have attempted to examine the effect of the administration of prebiotics and probiotics (synbiotics) simultaneously on ameliorating metabolic endotoxemia (Fernandes et al., 2017). Serum LPS or LPS binding protein (LPB) concentrations were the main outcomes measured in those trials. Measuring LPS levels before and after interventions can provide insights into the effectiveness of synbiotics in reducing inflammation and improving gut health.

## Discussion

Recently, Krumbeck et al. (2018) examined the effect of specific strains (*B.adolescentis* and *B.animals*) of *Bifidobacterium* along with galactooligosaccharides on intestinal permeability and endotoxemia among obese individuals. Metabolic markers, intestinal permeability, and endotoxin concentrations were measured in the study. This synbiotic treatment, composed of *Bifidobacterium* and galactooligsacchrides, enhanced the colonic permeability. During the study period, serum endotoxin was measured thrice. The authors concluded that no statistical significance was found in serum LPS and LBP among all the groups. However, the main drawback of the study is that the serum values of LPS and LBP were not presented either in the main manuscript or in the additional files section. It is unknown whether the baseline values of LPS and LBP are at high or low concentrations. Therefore, comparing the results of LPS and LBP with other clinical trials that used galactooligosaccharides or *Bifidobacterium* could not be applicable. Moreover, Krumbeck et al. (2018) did not consider measuring serum cytokines in the studied subjects. On the other hand, galactooligosaccharide supplementation led to a significant reduction in plasma LPS and C-reactive protein among obese individuals after 14 days of consumption (Morel et al., 2015). In addition, overweight and obese females who consumed *Bifidobacterium* for 8 weeks had lower levels of serum endotoxin compared with the control group (Gomes et al., 2017). Furthermore, compelling evidence suggests that prebiotics possess the capacity to bind with LPS within the intestinal lumen, effectively suppressing the translocation of LPS into the bloodstream (Snelson et al., 2021). Certain probiotic strains, particularly those belonging to the *Lactobacillus* and *Bifidobacterium* genera, assist in restoring the integrity of the gut barrier and reduce intestinal permeability. By enhancing the production of tight junction proteins, probiotics can strengthen the barrier function of the intestinal lining, thereby limiting the translocation of LPS from the gut lumen into the blood circulation (Han et al., 2016).

## Conclusion

In conclusion, the endotoxin findings by Krumbeck and her colleagues (2018) should be published in a specific corrigendum to illustrate the serum values of LPS and LBP. However, we suggest a deep discussion on the ineffectiveness of galactooligosaccharide over serum endotoxin levels and other inflammatory cytokines. Overall, this study is groundbreaking but further trials and testing are indispensable.
